# Analysis of Vascular Development in the *hydra* Sterol Biosynthetic Mutants of Arabidopsis

**DOI:** 10.1371/journal.pone.0012227

**Published:** 2010-08-17

**Authors:** Margaret Pullen, Nick Clark, Fatemeh Zarinkamar, Jennifer Topping, Keith Lindsey

**Affiliations:** The Integrative Cell Biology Laboratory, School of Biological and Biomedical Sciences, Durham University, Durham, United Kingdom; University of Oxford, United Kingdom

## Abstract

**Background:**

The control of vascular tissue development in plants is influenced by diverse hormonal signals, but their interactions during this process are not well understood. Wild-type sterol profiles are essential for growth, tissue patterning and signalling processes in plant development, and are required for regulated vascular patterning.

**Methodology/Principal Findings:**

Here we investigate the roles of sterols in vascular tissue development, through an analysis of the *Arabidopsis* mutants *hydra1* and *fackel*/*hydra2*, which are defective in the enzymes sterol isomerase and sterol C-14 reductase respectively. We show that defective vascular patterning in the shoot is associated with ectopic cell divisions. Expression of the auxin-regulated *AtHB8* homeobox gene is disrupted in mutant embryos and seedlings, associated with variably incomplete vascular strand formation and duplication of the longitudinal axis. Misexpression of the auxin reporter *proIAA2∶GUS* and mislocalization of PIN proteins occurs in the mutants. Introduction of the ethylene-insensitive *ein2* mutation partially rescues defective cell division, localization of PIN proteins, and vascular strand development.

**Conclusions:**

The results support a model in which sterols are required for correct auxin and ethylene crosstalk to regulate PIN localization, auxin distribution and *AtHB8* expression, necessary for correct vascular development.

## Introduction

The evolution of vascular tissues has been a critical event in the movement of plants from water to land, and in the construction of the higher plants. As well as providing mechanical strength, these tissues act as conduits for the transport of water, nutrients, hormones and even small RNA molecules around the plant [1]. The mechanisms by which the formation and maintenance of the patterns of vascular tissues are regulated are still poorly understood at the molecular level [2], [3]. It is in the embryo that the establishment of the early vascular tissues, the procambium, occurs. In *Arabidopsis thaliana*, this process involves stereotypical and predictable patterns of cell division, expansion and differentiation coordinated by signalling systems, and notably auxin, to execute spatially and temporally controlled patterns of gene expression [4], [5].

A number of models have been proposed to account for vascular development. Sachs pioneered the ‘auxin signal flow canalization hypothesis’, which suggests that polar auxin transport promotes strand extension [6]. Scarpella *et al*. [7] extended this model to suggest that expression domains of the auxin efflux carrier-encoding *PINFORMED1* (*PIN1*) [8] in the epidermis provide positional information for the specification of procambial cells and the positioning of veins in the leaf. Inhibition of polar auxin transport by chemical inhibitors has also been shown to affect leaf vein patterning [9], further implicating auxin transport in vascular patterning.

Mutant screens have led to the identification of several classes of gene that are required for wild-type vascular development. The *monopteros* mutant, for example, is expressed in the Arabidopsis embryo, and the mutant is characterized by abnormal divisions in the prospective procambial tissue in the embryo and defective cotyledonary vein formation [10], [11]. Interestingly, the MONOPTEROS (MP) protein is a member of the auxin response factor (ARF) family, a class of transcription factors that regulate the transcription of auxin-responsive genes [12]. MP interacts with the related NONPHOTOTROPIC HYPOCOTYL4 (NPH4) [13]. ATHB8 is an HD-Zip protein which is a positive regulator of vascular cell differentiation, and its overexpression can lead to excessive xylem cells in vascular bundles [14], [15]. Recent data show that *ATHB8* expression is regulated directly by MP, is required for procambial cell specification, and its loss of function phenotype is masked by MP function [16]. A related protein is PHAVOLUTA, also an HD-Zip transcription factor that is required for vascular cambium development as well as other aspects of leaf morphogenesis [17], [18]. Other mutant analyses provide alternative models for the control of vascular patterning to the auxin flow canalization model [19]. Recently, Petricka *et al*. [20] identified 45 different loci in a screen for mutants affecting vein pattern in Arabidopsis.

One intriguing class of mutants that show defective vascular patterning is that defective in sterol biosynthesis. This includes *orc/sterol methyltransferase1 (smt1)/cephalopod* (*cph*), *hydra1* (*hyd1*), *fackel* (*fk*)*/hyd2*, *cotyledon vascular pattern* (*cvp1/smt2*) and *cyclopropylsterol isomerase1-1* (*cpi1-1*) [21]–[27]. Although dwarfed, these can be considered as being distinct from the brassinosteroid (BR) *dwarf* mutants, even though they are defective in enzymes upstream of BR synthesis. For example, they exhibit defective embryonic and/or seedling cell patterning, including vein patterning, are typically seedling-lethal, and cannot be rescued by exogenous application of BRs [28]. Metabolic profiling of sterol methyltransferase mutants similarly suggests that developmental defects in these mutants are not due to defective BR content [29].

This raises the interesting question of the role of sterols (as distinct from BRs) in plant development. It has been postulated that specific sterols that are absent from, or are present at abnormally low levels in, the mutants and are required for appropriate signalling for cell division and expansion. Schrick *et al*. [23], for example, following an analysis of the *fk* mutant, propose a model in which specific sterol molecules, distinct from BRs, may have specific signalling roles required for correct cell patterning. Studies on *fk* have shown that a range of novel sterols are produced in these mutants [24], and various sterol intermediates accumulate to abnormal levels [22]. Any of these components might interfere with endogenous sterol-mediated signalling systems, and so disrupt development [30].

Since sterols are components of cell membranes, it is also possible that at least some of the developmental defects are the consequence of aberrant membrane function, such as altered membrane permeability and/or fluidity. Modified sterol profiles might also cause aberrant localization or function of important membrane-bound proteins such as receptors or transport proteins. Support of this hypothesis comes from the analysis of several sterol synthesis mutants. Both *fk^hyd2^* and *orc* show mis-expression of the *DR5::GUS* auxin reporter [26], [31], and the *hyd/fk* mutants show enhanced auxin responses [26]. Inhibition of the auxin influx carrier AUX1 by 1-naphthoxyacetic acid (1-NOA) failed to block these responses in *hyd* mutants. This suggests either that the AUX1 protein is by-passed, perhaps due to an increased membrane permeability to auxin; or the mutants exhibit an increased activity of the AUX1 protein that is not inhibited by 1-NOA, perhaps due to a conformational change. In the *hyd* mutants, PIN3 localization showed a proximal shift to the columella initials at day 9 post-germination, then disappeared, associated with the loss of identity of the columella in these mutants [26]. In *orc*, the application of low concentrations of the AUX1-dependent auxin 2,4-D led to rescue of trichoblast polarity, suggesting that while auxin influx does not appear to be defective, auxin response or availability might be [31]. Rates of polar auxin transport in *orc* were reduced significantly compared to wild-type, and although AUX1 positioning was normal in the mutant, the PIN1 and PIN3 proteins were mis-localized. More recently, Men *et al*. [27] showed that defective sterol profiles in the *cpi1-1* mutant are associated with defective PIN2 endocytosis and polar localization following cytokinesis. Furthermore, Pan *et al*. [32] showed that sterols are required for auxin-mediated PIN2 endocytosis; and Carland *et al*. [29] have found that sterol methyltransferase (*smt*) mutants of *Arabidopsis* exhibit a range of auxin-mediated responses, independent of BR function.

A view is therefore emerging to suggest that sterols are required for correctly regulated auxin signalling, by mediating carrier protein localization or functionality in the membrane. Further evidence for altered signalling comes from the observation that the *hyd/fk* mutants may be defective in the ethylene response pathway [26], [33]. However, the link between sterols, auxin and ethylene remains unclear. Recent work shows that, in the root, ethylene can induce auxin biosynthesis and transport [34], [35], and we have recently developed a mathematical model to describe auxin, ethylene and cytokinin interactions in the Arabidopsis root [36]. Consistent with this, aspects of defective auxin signalling and auxin-dependent root cell patterning and growth can be rescued in the *hyd/fk* mutants by the inhibition of ethylene signalling [33]. CONSTITUTIVE TRIPLE RESPONSE-1 (CTR1) acts as a repressor of auxin biosynthesis in *Arabidopsis* [37]. In the constitutively ethylene responsive *ctr1* mutant, the local distribution of auxin regulating the establishment of cell polarity is disrupted, providing further evidence for a link between auxin and ethylene signal transduction.

To investigate the link between sterols, auxin and ethylene in vascular development, we studied these relationships in the sterol biosynthesis mutants *hyd1* and *fk^hyd2^*. These genes encode adjacent enzymatic steps in the sterol biosynthetic pathway and their loss of function mutants have similar phenotypes. We found that defects in cell division, auxin transport machinery and vascular development were evident in the mutants, but those associated with auxin transport and responses in particular could be rescued to a significant extent by the inhibition of ethylene signalling.

## Results

### Abnormal vascular patterning is associated with abnormal cell division in the hyd/fk mutants

**Figure 1:**
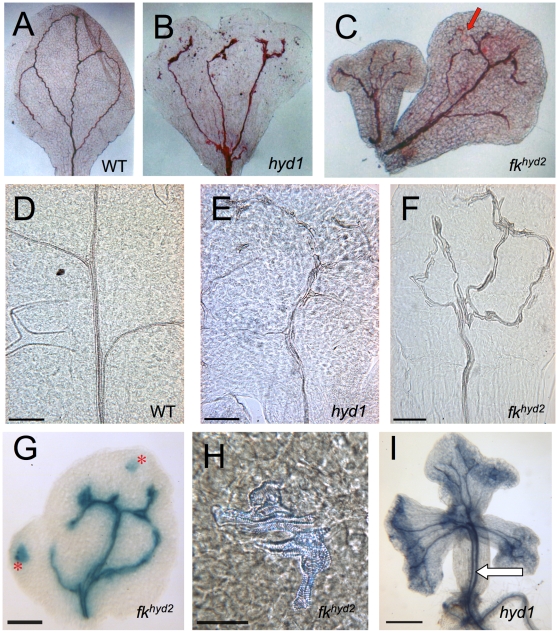
Vascular strand defects in *hyd/fk* mutant seedlings. A: Safranine-stained wild-type cotyledon showing the tissue pattern of vascular differentiation, ×30 magnification. B: Safranine-stained *hyd1* cotyledon, with dissociation of the primary vasculature into three traces, ×30 magnification. C: Safranine-stained cotyledons from a *fk^hyd2^* seedling, with primary vascular dissociation in one of the cotyledons at the point indicated by the arrow, ×30 magnification. D–F: Cleared tissues from the central lamina of the first true leaf of 12 dpg plants of wild-type (d), *hyd1* (e), *fk^hyd2^* (f); bars  = 200 µM. G: *fk^hyd2^* cotyledon showing *proPIN1::GUS* expression to reveal vascular strands and ‘islands' of vascular tissue (asterisks); bar  = 250 µM. H: Vascular ‘island' of disconnected xylem cells in *fk^hyd2^* cotyledon (7 dpg) cleared with chloral hydrate; bar  = 50 µm. I: Aniline blue-stained *hyd1* seedling (8 dpg) showing duplicated vascular strand in the mutant (arrow) ; bar  = 500 µM.

In both *hyd1* and *fk^hyd2^* seedling cotyledons, a varied vascular pattern is apparent as incomplete and isolated sections of xylem within the defined provascular field (Figs. 1A–H). Xylem ‘islands' are evident (Figs. 1G,H), indicative of a low level of co-ordination of cell patterning and differentiation. The main strands also show variable vessel size and orientation, with cells running parallel with each other, or contorted into a varied strand morphology not seen in wild-type plants. Vascular defects in the hypocotyl include dissociation of the vascular trace above the primary branch point beneath the SAM, and in some individuals, a duplication of the entire longitudinal axis (Fig. 1I). No examples of axis duplication were observed in the wild-type.

Aniline blue staining was carried out to reveal phloem-associated callose in cotyledons and true leaves. The results show that, whereas in wild-type seedlings the xylem and phloem traces are closely aligned (Supplementary Fig. S1A), in the *hyd* mutants callose accumulates ectopically, and is variably associated with xylem traces showing a lack of coordinated differentiation of the two vascular cell types (Supplementary Figs. S1D–F).

**Figure 2:**
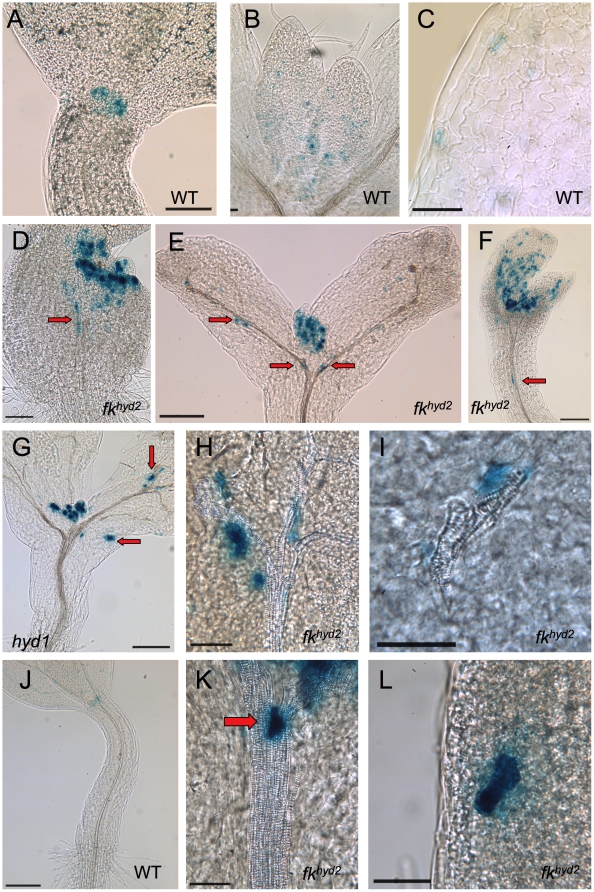
*proCYC1At::CDB::GUS* expression in *hyd/fk* seedlings. A: Wild-type hypocotyl-cotyledon junction, 3 dpg; bar  = 100 µm. B: Wild-type leaf primordia, 7 dpg; bar  = 100 µm. C: Wild-type cotyledon epidermis, 7dpg; bar  = 50 µm. D: *fk^hyd2^* hypocotyl-cotyledon junction, 3 dpg. Arrow indicates ectopic cell division events in hypocotyl and cotyledon; bar  = 100 µm. E: *fk^hyd2^* hypocotyl-cotyledon junction, 7 dpg. Arrows indicate ectopic cell division events in hypocotyl and cotyledon; bar  = 200 µm. F: *hyd1* hypocotyl-cotyledon junction, 3 dpg. Arrow indicates ectopic cell division events in hypocotyl; bar  = 100 µm. G; *hyd1* hypocotyl-cotyledon junction, 7 dpg. Arrows indicate ectopic cell division events in hypocotyl and cotyledon; bar  = 200 µm. H, I: *fk^hyd2^* cotyledons at 7 dpg, showing expression in proximity to dissociated or disjunct xylem vessels; bar  = 50 µm. J: Wild-type hypocotyl region of a 3 dpg seedling, showing expression restricted to stomatal precursors of the cotyledon epidermis and the developing first pair of true leaves; bar  = 100 µm. K: *fk^hyd2^* hypocotyl (3 dpg) showing expression in association with the hypocotyl stele (arrow); bar  = 100 µm. L: *fk^hyd2^* hypocotyl epidermis (3 dpg) showing mis-oriented ectopic cell division event spanning two longitudinal cell files; bar  = 50 µm.

The mutant seedlings also exhibit ectopic cell division in the shoot associated with defective vascular patterning, as visualized by the expression of *CYC1At::CDB::GUS*; this reporter marks cells entering mitosis [38]. In wild-type aerial parts, expression of this marker is confined predominantly to the young leaf primordia and developing stomata (Figs. 2A–C). In the 3 dpg shoot apices of *fk^hyd2^* (Fig. 2D) and *hyd1* (Fig. 2F), cotyledons show variable patterns and levels of *CYC1At::CDB::GUS* expression. Young mutant leaf primordia (as in Figs. 2E, G) show a spread of division activity across the SAM-containing region. By 7 dpg, strong reporter expression is retained around the SAM and in young primordia of both mutants (beyond the regions that express the gene in wild-type). In cotyledons, ectopic GUS activity is reduced, but is seen at foci associated with discontinuities in the xylem strands (Figs. 2G–I).

No expression of *CYC1At::CDB::GUS* was observed in hypocotyls of 3–7 dpg wild-type seedlings (Fig. 2J). However, in *hyd/fk* mutant hypocotyls there was seen cell division events in the stele of the upper hypocotyl in the vicinity of branching points in the xylem vascular strands (Fig. 2K) and in the hypocotyl epidermis at 7 dpg (Fig. 2L). The ectopic division event highlighted in Fig. 2L is associated with a cell expanding at an oblique angle to the cell file orientation in this region, indicative of defects in the control of axial cell elongation.

### *hydra* mutants exhibit defective *AtHB8* gene expression

To investigate the link between defective sterol profiles and the establishment of vascular patterning in the embryo, expression of the auxin-regulated *proAtHB8∶GUS* reporter was analysed in the *hyd1* and *fk^hyd2^* mutant backgrounds. The HD-Zip transcription factor AtHB8 is a positive regulator of vascular cell differentiation; its promoter is active in cells prior to their adopting vascular procambial cell fate, as well as in developed strands [14], [16]. It is the earliest known marker of vascular development [39].

**Figure 3:**
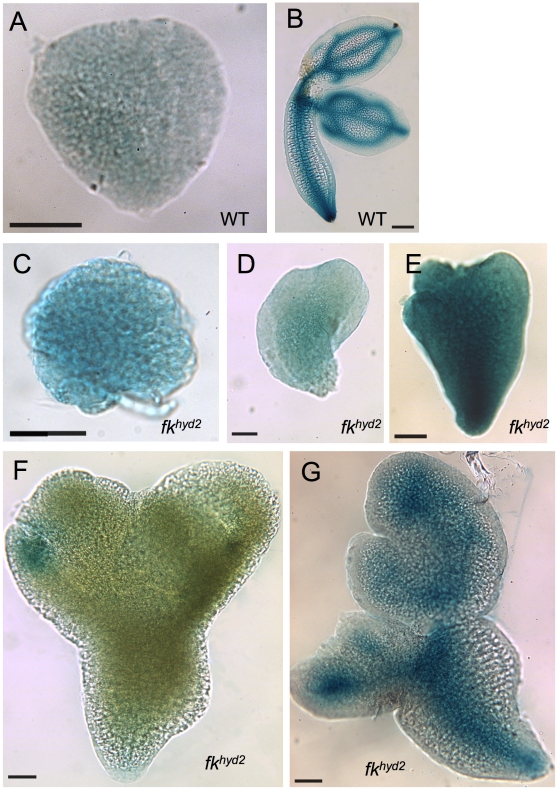
*proAtHB8∶GUS* expression is disorganized in *fk^hyd2^* embryos. A: Wild-type globular embryo, showing very low levels of expression; bar  = 50 µm. B: Wild-type older torpedo-stage embryo, showing expression in procambium; bar  = 100 µm. C: *fk^hyd2^* globular-stage embryo, showing stronger expression than wild-type embryos at the same stage (a); bar  = 50 µm. D: *fk^hyd2^* heart-stage embryo; bar  = 50 µm. E: *fk^hyd2^* torpedo-stage embryo; bar  = 50 µm. F: *fk^hyd2^* early torpedo-stage embryo, showing highly localized expression in cotyledonary tissue; bar  = 50 µm. G: *fk^hyd2^* late torpedo-stage embryo, showing expression in presumptive procambium; bar  = 50 µm.

Fig. 3 shows the expression of *proAtHB8∶GUS* in wild-type and *fk^hyd2^* mutant embryos. In the wild-type, GUS activity is barely detectable during the globular-heart stage transition (Fig. 3A), and then resolving to the procambial traces in the pro-cotyledons, hypocotyl and root of older embryos (Fig. 3B).

In *fk^hyd2^* mutant embryos (which show a very similar expression pattern to *hyd1* mutant embryos; not shown), a more variable expression pattern of *proAtHB8∶GUS* activity is seen, associated with a variable morphology between siblings (Figs. 3C–G). Analysis at the heart stage transition reveals diffuse GUS activity throughout the embryo (Fig. 3C), whilst later stages exhibit variable levels of signal - either relatively low (Fig. 3D, F) or relatively high (Fig. 3E). More mature embryos may have a rudimentary procambial trace, although may not demonstrate normal patterning (Fig. 3G). These observations suggest that the patterning processes allowing the definition of the procambial strands have been disrupted in the *hyd/fk* mutants.

**Figure 4:**
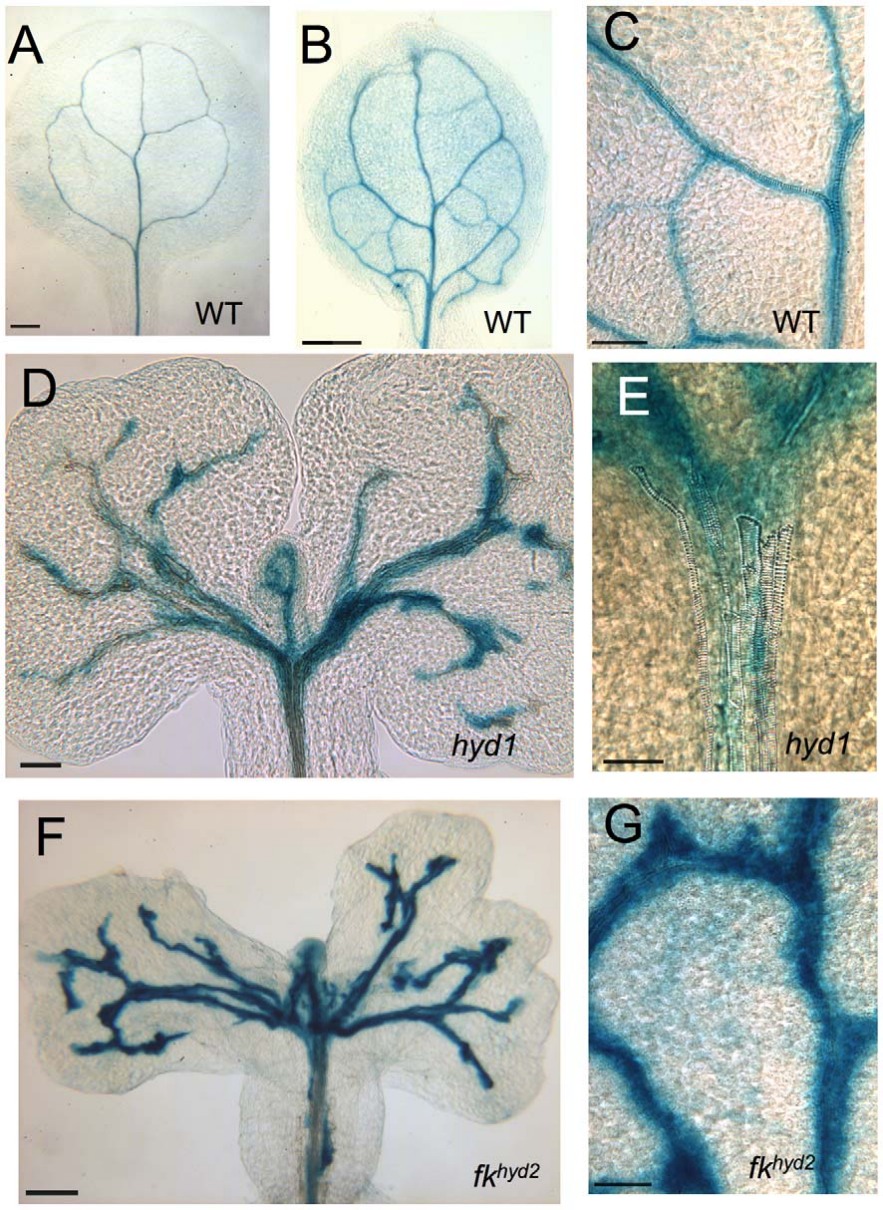
*proAtHB8∶GUS* expression in *hyd/fk* seedlings. A: Wild-type cotyledon; bar  = 100 µm. B: Wild-type true leaf; bar  = 250 µm. C: Vascular trace in wild-type true leaf (detail from b); bar  = 50 µm. D: *hyd1* seedling (3 dpg); bar  = 50 µm. E: *hyd1* seedling (detail from d) showing lack of procambial and vascular strand coordination at the branch point in the upper hypocotyl; bar  = 50 µm. F: *fk^hyd2^* cotyledons 7 dpg; bar  = 250 µm. G: Detail of vascular strand from *fk^hyd2^* leaf (7 dpg), showing stronger expression than in wild-type vascular trace (c); bar  = 50 µm.

Variability of *proAtHB8∶GUS* activity is also seen in the mutant seedlings (Fig. 4), reflecting disorganization of vascular strands. In cotyledons of *fk^hyd2^* in particular, the intensity of *proAtHB8∶GUS* activity is relatively high compared with wild-type (compare Fig. 4C with Fig. 4G).

### *hyd/fk* mutants show altered *proIAA2∶GUS* expression

Auxin is a known regulator of vascular patterning, acting in part at least via ATHB8 [16], and the vascular defects in the *hyd/fk* mutants may suggest defects in auxin transport, localization and response, linked to altered *proAtHB8∶GUS* expression. To investigate this, we monitored the expression pattern of *proIAA2∶GUS* in the *hyd/fk* mutant aerial parts. *IAA2* is an early auxin response *AUX/IAA* gene, induced strongly and specifically by endogenous auxin [40], [41]. The *proIAA2∶GUS* reporter [42] therefore acts as a marker for early auxin-induced gene expression and indicates the presence of active auxins and auxin responses. Previously, we showed that *proIAA2∶GUS* expression declined in *hyd/fk* mutant root tips after ca. 18 dpg [33] but had no information on expression in shoots.

**Figure 5:**
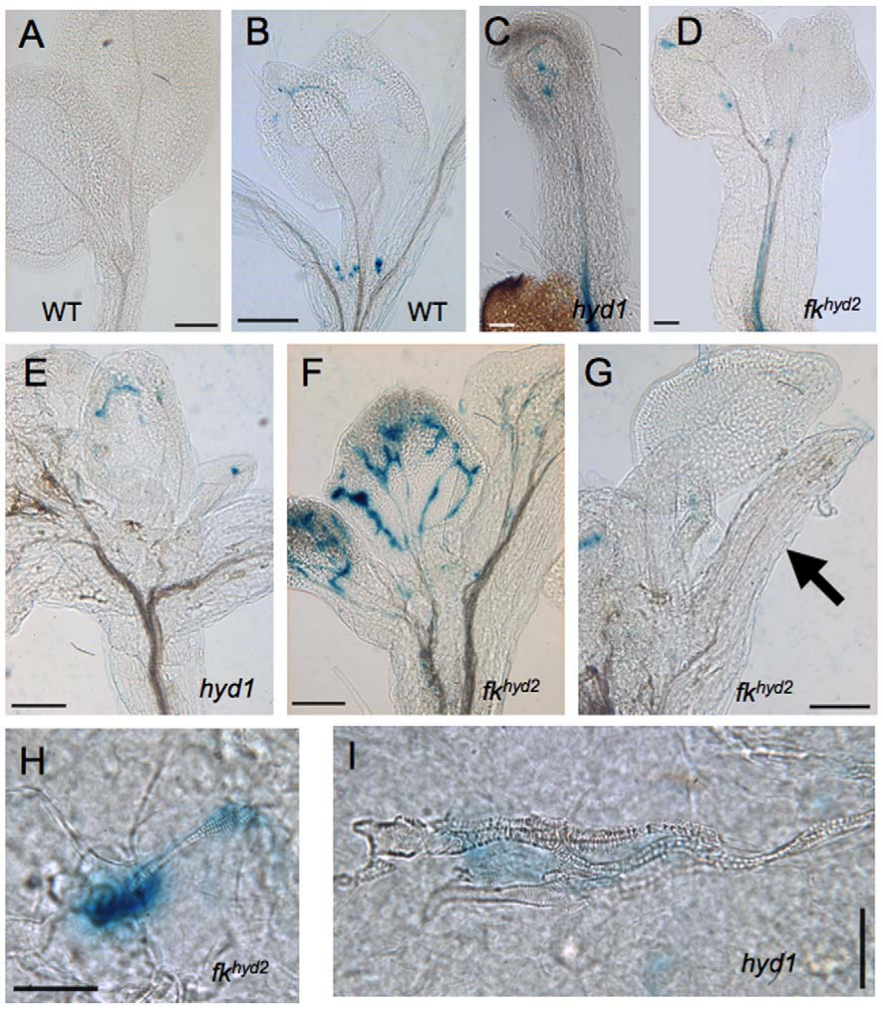
*proIAA2∶GUS* expression is disorganized in *hyd/fk* seedlings. A: Wild-type seedling (3 dpg), showing absence of expression; bar  = 200 µm. B: Wild-type seedling (12 dpg), showing expression in stipules and low levels in vascular traces; bar  = 200 µm. C, D: *hyd1* (c) and *fk^hyd2^* (d) seedlings (3 dpg), showing ectopic expression in the cotyledon and hypocotyl developing vasculature; bar  = 200 µm. E, F: *hyd1* (e) and *fk^hyd2^* (f) seedlings (12 dpg), showing ectopic expression in the cotyledon developing vasculature; bar  = 200 µm. G: *fk^hyd2^* seedling (12 dpg), showing lack of *proIAA2∶GUS* expression in the vasculature of the radialized leaf (arrow); bar  = 200 µm. H, I: *proIAA2∶GUS* expression associated with disjunct and dissociated xylem from *hyd1* cotyledons (12 dpg); bar  = 50 µm.

In the wild-type shoot, no *proIAA2∶GUS* expression is discernible in cotyledons (Fig. 5A). As true leaves begin to emerge between 5 and 7 dpg, a stipule signal appears, followed by transient definition of the leaf vascular traces, prior to differentiation of the xylem vessels (Fig. 5B). Seedlings of both *hyd1* and *fk^hyd2^* at 3 dpg each show ectopic expression patterns of *proIAA2∶GUS* in shoot tissues. GUS activity, confined to the young root stele in wild-type, extends part-way into the mutant hypocotyl stele, and appears as a localized ectopic signal in the cotyledons (Figs. 5C, D). In older *hyd/fk* shoot tissues, ectopic expression in the cotyledon occurs in the vicinity of late-differentiating xylem, vascular islands, and in regions where the xylem trace shows poor coherence in the strand, particularly at the hydathodes (Figs. 5E, F). This ectopic expression was present in most cotyledons, although no signal was found in radialized cotyledon structures (Fig. 5G). In true leaves, both *hyd/fk* mutants show an enhanced *proIAA2∶GUS* expression in the vascular traces during lamina development (Figs. 5H, I). While recognizing that histological GUS activity is only semi-quantitative, these observations suggest that auxin is poorly localized in the developing vasculature of young leaves, possibly synthesized but not efficiently exported from these cells, consistent with aberrant *proAtHB8∶GUS* expression.

### *hydra/fk* mutants show defective polar auxin transport machinery

The PIN-FORMED (PIN) family of proteins is required for auxin efflux from cells, and control directionality of auxin flow [43], which is required for the control of vascular patterning [7]. However, the interdependence of directional auxin transport with ethylene signalling and sterols is not well defined. To understand this better, we investigated whether an inhibition of ethylene signalling influenced polar auxin transport machinery in the *hyd*/*fk* mutants.

**Figure 6:**
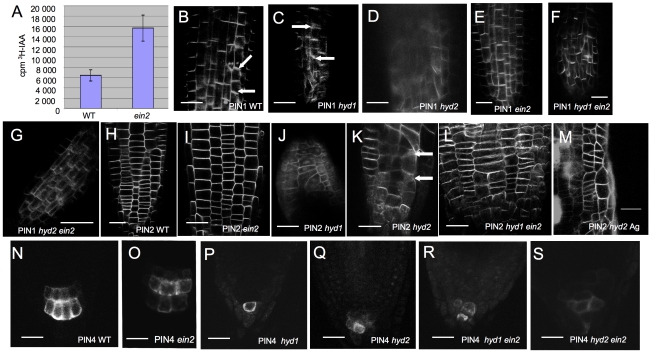
PIN localization is defective in *hyd/fk* seedlings. A: Polar auxin transport assays in wild-type and *ein2* hypocotyls. Bars represent standard errors, n = 8. B–G: PIN1∶GFP localization (arrows) in roots of wild-type (B), *hyd1* (C), *fk^hyd2^* (D), *ein2* (E), *hyd1 ein2* (F) and *fk^hyd2^ ein2* (G). Wild-type localization is predominantly at the basal end of the cells, but is less clearly localized in *hyd1* and *fk^hyd2^*. Bars  = B–F, 50 µM; G, 25 µM. H–M: PIN2∶GFP localization in roots of wild-type (H), *ein2* (I), *hyd1* (J), *fk^hyd2^* (K), *hyd1 ein2* (L) and *fk^hyd2^* treated with 10 mM silver thiosulphate (M). Bars  = 50 µM. N–R: PIN4∶GFP localization in roots of wild-type (N), *ein2* (O), *hyd1* (P), *fk^hyd2^* (Q), *hyd1 ein2* (R) and *fk^hyd2^ ein2* (S). Bars  = 20 µM.

Analysis carried out over a developmental time course reveals that, while wild-type PIN1∶GFP and PIN2∶GFP localization can occur correctly in many cells, the mutants show more cell-to-cell variability than in wild-type, and localization can be very diffuse (Fig. 6). Poor PIN2∶GFP localization was observed (Figs. 6J, K), associated with defective epidermal development in the mutants [21]. Similarly, PIN4∶GFP, expressed in the columella cells in wild-type [44], is also poorly expressed in both *hyd1* and *fk^hyd2^*, though localization is broadly as in wild-type (i.e. on all cell faces) in the cells in which expression is detectable (Figs. 6P, Q). The defective PIN localization analysed here in roots (due to ease of visualization) is consistent with a predicted requirement for sterols in vesicle transport and recycling [27], [31] and with the observed defective auxin distribution and meristem and vascular patterning [22], [26], [33].

Given that the inhibition of ethylene signalling partially rescues auxin responses and cell patterning in the root in the *hyd*/*fk* mutants [26], [33], we investigated the effects of *ein2* or silver treatment, both of which reduce ethylene responses, on PIN∶GFP localization. The *ein2* mutation conveys a systemic ethylene resistance through the elimination of a signalling relay step between the cytoplasm and the nucleus [45]. Analysis of ^3^H-IAA transport in hypocotyls shows *ein2* has an enhanced rate of polar auxin transport compared to wildtype, and has normal PIN localization (Fig. 6). The expression and localization of PIN1, PIN2 and PIN4 GFP fusion proteins exhibited the wild-type pattern both in the single *ein2* mutant or following silver treatment (Figs. 6E, 6I and 6O). Experimentally reduced ethylene responses in both *hyd1* and *fk^hyd2^* mutants led to a more ordered localization of PIN1∶GFP in particular (Figs. 6B–G), with partial rescue for PIN2∶GFP (Figs. 6H–M) but no rescue of PIN4∶GFP (Figs. 6N–S), compared with individual *hyd/fk* mutants. Consistent with these observations, ethylene inhibition leads to partial phenotypic rescue of root development in the *hyd*/*fk* mutants [33].

### Inhibition of ethylene responses in *hyd/fk* partially rescues vascular pattern

To investigate the effect of ethylene signalling on vascular tissue patterning in the *hyd/fk* mutants, *hyd1 ein2* and *fk^hyd2^ ein2* double mutants were analysed. Comparisons were also made with wild-type and *ein2* mutants.

**Figure 7:**
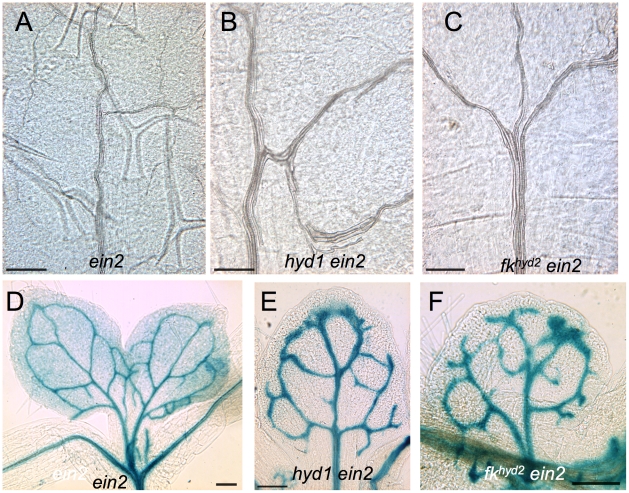
Vascular strand coherence is improved by the *ein2* mutation in the *hyd/fk* mutants. A–C: Cleared tissues from the central lamina of the first true leaf of 12 dpg plants of *ein2* (A), *hyd1 ein2* (B) and *fk^hyd2^ ein2* (C); bars  = 200 µm. D–F: *proAtHB8∶GUS* expression in *ein2* and *hyd/fk ein2* mutants. D: *ein2* true leaves of a 10 dpg seedling; bar  = 100 µm. E: *hyd1 ein2* first true leaf of a 10 dpg seedling; bar  = 250 µm. F: *fk^hyd2^ ein2* first true leaf from 10 dpg seedling; bar  = 200 µm.

The xylem traces of cotyledon primary vascular strands in *hyd/fk ein2* mutants are more coherent than observed in *hyd/fk* single mutants, and less ‘noise' is evident within the xylem (Figs. 7A–C, cf. Fig. 1). Figs. 7D–F show seedlings of *ein2* mutants and *fk^hyd2^ ein2* double mutants expressing the *proAtHB8∶GUS* procambial cell identity reporter. The activity of this transgene in *ein2* seedlings (Fig. 7D) is indistinguishable from wild-type (cf. Figs. 4A–C), and the patterns of differentiating xylem traces in wild-type and *ein2* show no differences. The *hyd 1 ein2* and *fk^hyd2^ ein2* seedlings (Figs. 7E, F) have greater vascular strand coherence than do *fk/hyd* single mutants (cf. Figs. 4D–G).

**Figure 8:**
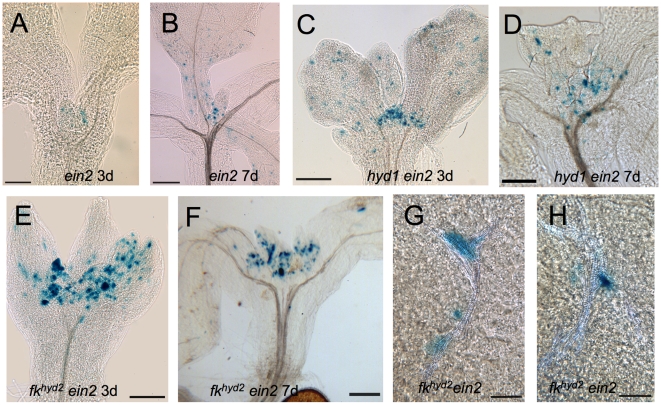
Inhibition of ethylene signalling in *hyd/fk* mutants leads to partial rescue of cell division patterning. A–H: *proCYC1At::CDB::GUS* expression in *ein2* mutants and *hyd/fk ein2* double mutants. A, B: *ein2* seedlings at 3 dpg (A) and 7 dpg (A); bars  = 100 µm (A), 200 µm (B). C, D: *hyd1 ein2* double mutant seedlings at 3 dpg (C) and 7 dpg (D); bars  = 200 µm. E, F: *fk^hyd2^ ein2* double mutant seedlings at 3 dpg (E) and 7 dpg (F); bars  = 200 µm (E), 250 µm (F). G, H: Detail of *fk^hyd2^ ein2* double mutant cotyledons at 7 dpg, showing that ectopic cell division activity persists in the vicinity of dissociated and disjunct xylem vessels; bars  = 50 µm.

Introduction of the *ein2* mutation into the *hyd1* background led to a decreased frequency of ectopic cell divisions in the shoot at 7 dpg, though not at 3 dpg, as monitored by *CYC1At::CDB::GUS* expression (Fig. 8). In contrast to both *hyd1 ein2* and *ein2* (Figs. 8A–D), the *fk^hyd2^ ein2* shoot apices show relatively high levels of *CYC1At::CDB::GUS* expression at 3 dpg and 7 dpg (Figs. 8E, F). Furthermore, ectopic cell division events in *fk^hyd2^ ein2* double mutants were found in similar positions as in *fk^hyd2^* single mutants, in association with compromised xylem integrity, vascular islands and in association with late-differentiating xylem (Figs. 8G, H). Therefore, repression of ethylene signalling in the *hyd1* mutants partially inhibits the ectopic cell divisions seen in the mutant shoots, but no effect was evident in the more severe *fk^hyd2^* mutant.

**Figure 9:**
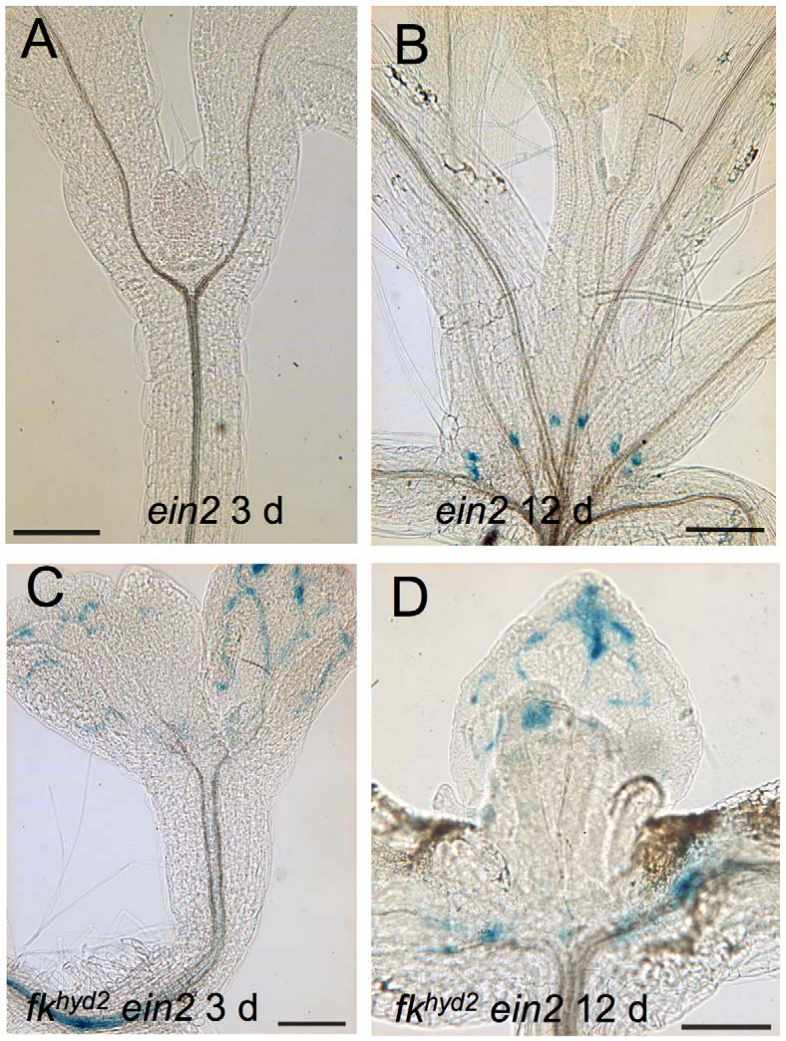
Inhibition of ethylene signalling on *proIAA2∶GUS* expression in *fk^hyd2^* mutants. A–D: *proIAA2∶GUS* expression in *ein2* and *fk^hyd2^ ein2* mutants. A, B: *ein2* seedling at 3 dpg (a) and 12 dpg (b); bars  = 200 µm. C,D: *fk^hyd2^ ein2* seedling at 3 dpg (C) and 12 dpg (D) showing similar pattern but reduced intensity of expression compared to *fk^hyd2^* single mutants (see Figs. 5D, G, F); bars  = 200 µm.

The *hyd/fk* mutants were previously reported to have enhanced ethylene signalling [26], [33]. The auxin-responsive *proIAA2∶GUS* reporter was used to determine whether ethylene signalling was responsible for the altered auxin localization or responses in aerial parts of *fk^hyd2^ ein2* double mutants. Expression of the *proIAA2∶GUS* auxin-responsive reporter showed similar positional signals in wild-type and *ein2* single mutant seedlings between 3 and 12 dpg (Figs. 9A, B cf Fig. 5), suggesting that auxin positional localization functions normally in *ein2*. The activity of *proIAA2∶GUS* in *fk^hyd2^ ein2* mutant cotyledons (as distinct from the true leaves) appeared similar to that in *fk^hyd2^* single mutants, and likewise occurred in association with xylem disjunctures (Fig. 9C). However, *fk^hyd2^ ein2* seedlings showed *proIAA2∶GUS* expression that was more clearly defined in association with vascular tissues in true leaves (e.g. Fig. 9D). Similar results were found for *hyd1 ein2* seedlings (data not shown). These results suggest that poor auxin localization in post-embryonic aerial parts of the *hyd/fk* mutants is in part dependent on ethylene defects, i.e. apparent in leaves but not the embryonically derived cotyledons. *EIN2* is not strongly expressed in the embryo [5], and might not therefore be expected to rescue these embryonically derived structures.

## Discussion

Vascular tissue development is regulated by complex interactions between multiple signalling pathways, and the nature of the interactions is far from clear, though progress is being made in defining the mechanisms involved [3]. A role for sterols as essential components of vascular patterning is evident from biosynthesis mutant phenotypes, but the molecular basis of their actions is not well understood. We have investigated sterol-hormone interactions through the analysis of double mutants in sterol synthesis and ethylene responses, and monitored effects on PIN proteins and auxin-regulated genes, including a key gene, *AtHB8*, which is an auxin-regulated transcription factor required for vascular development.

The *hyd1* and *fk^hyd2^* sterol mutants are similarly defective in several aspects of development. Vascular coordination is poor throughout the mutant seedling and originates from patterning problems at the point of procambial coordination. Embryonically derived tissues such as cotyledons generally showed more defective vascular patterning than did true leaves. Disjunct and dissociated xylem vessels were seen typically in association with persistent ectopic cell division activity. These phenotypes are distinct from BR mutants which are dwarfed and less severely affected in cellular patterning and histogenesis; though the BR signalling pathway is required for wild-type vascular differentiation [46]–[48].

There is a growing body of evidence that correct sterol profiles are necessary for the controlled integration of plant hormone signalling. We have shown previously that the *hyd1* and *fk^hyd2^* mutants exhibit defects in both auxin and ethylene signalling [26], [33]. The pharmacological or genetic inhibition of ethylene signalling can restore to a significant degree the cellular organization and activity of the root meristems and, as we now show here, vascular tissues. Ethylene signalling inhibition also led to a restoration of auxin-mediated gene expression patterns and the localization of PIN1 and PIN2, though not the consistently poor production of PIN4. This supports the concept of cross-talk between sterols, ethylene and auxin.

Carland *et al*. [25] showed that the *cvp1* mutant, defective in the enzyme sterol methyltransferase 2 (SMT2), has misshapen and misaligned vascular cells, as well as abnormal organ expansion and elongation. The authors proposed that SMT2 may be required to establish a polarizing signal necessary for wild-type vascular patterning. In support of this, the same group recently found that *smt* mutants exhibited defects in auxin responses and localization [29]. Previously we found that the *hyd* mutants show defective PIN3 localization in the root tip [26], and Willemsen *et al*. [31] found that the *orc* allele of *SMT1* exhibits defective PIN1 and PIN3 localization. In addition, abnormal expression of the *proIAA2∶GUS* reporter was found in the roots of *hyd1* and *fk^hyd2^* seedlings [33]. Since root meristem function depends on controlled auxin distribution and auxin-mediated gene expression [49], [50], these observations suggest that the mis-direction and hence mis-localization of auxin at the root apex of sterol mutants can account for their previously reported defective root meristem function [21], [26], [31], [33]. These data implicate a role for sterols in regulating cell polarity and auxin distribution. In support of this view, Grebe *et al*. [51] have evidence that sterol and PIN2 recycling share a common endosomal pathway, and that PIN2 localization, and auxin-mediated inhibition of PIN2 endocytosis is inhibited in mutants with defective sterol profiles [27]. Pan *et al*. have also shown that the *fk* mutant has defective PIN2 recycling [32].

In this paper we show that the *hyd1* and *fk^hyd2^* mutants show cell-to-cell variability in the localization of PINS 1, 2 and 4, and this can be partially rescued by the inhibition of ethylene signalling. PIN1 is known to have a major role in shoot auxin translocation in addition to auxin transport into the root tip [8], [52]. We show defects in *proIAA2∶GUS* expression in the mutant aerial tissues, indicating an altered patterning of auxin distribution and/or responses. One interpretation of the persistence of ectopic *proIAA2∶GUS* activity in the vicinity of discontinuities in the vascular xylem is that auxin transport is compromised by a lack of coherence between vessel elements, allowing a local and ectopic accumulation of auxin. It is also possible that incorrect auxin localization, due to abnormal PIN protein function, in turn causes defective vascular patterning. Evidence in favour of this latter hypothesis is supported further by the misexpression of *proAtHB8∶GUS*. This transcription factor is auxin-regulated, and is a positive regulator of vascular differentiation in Arabidopsis [14]–[16]. Here we show that the *proAtHB8∶GUS* marker is very poorly localized in the *hyd* mutants, particularly in embryos where longitudinal procambial traces are established which define the plant body, and also in cotyledons and true leaves of establishment-stage seedlings.

Correct vascular patterning is also a product of the coordination of cell expansion and cell division. These processes are severely disrupted in *hyd1* and *fk^hyd2^* and other sterol mutants, though a number of genes affect vascular strand development [19], [20]. Similarly, the sterol-deficient *cvp1* has reduced axial cell expansion [25], and Schrick *et al*. [53] have shown that sterol mutants exhibit cell wall stubs, indicative of defective cytokinesis. These authors proposed that sterols are required for cellulose biosynthesis and cell wall construction; one possible mechanism might be via a role for sitosterol as an initiating factor in cellulose biosynthesis [54]. The *hyd* mutants exhibit ectopic lignin and callose accumulation (Fig. S1), showing a dissociation between cell patterning and wall biochemistry. It is also possible that defects in phragmoplast formation in these mutants contributes to, or is associated with, the observed defective PIN protein localization.

The role of sterol interactions with ethylene signalling and auxin is intriguing. The *hyd* mutants show enhanced ethylene responses, as well as defects in auxin responses [26], [33]. Recent work provides evidence that ethylene can induce auxin biosynthesis and transport in the root, a basis for crosstalk mechanisms [34]–[36], [55]. In addition, enhanced ethylene responses in the root tip can lead not only to reduced root cell elongation, but also to ectopic divisions of the quiescent centre cells [56]. Similarly, ethylene can promote aberrant divisions in the shoot [57]. Given that we have demonstrated partial rescue by *ein2* of auxin-mediated gene expression and vascular patterning in the *hyd* mutants, it is possible that sterol-mediated ethylene signalling defects, perhaps as a result of a (currently obscure) sterol dependence of ethylene signalling components [28], results in defective auxin signalling or distribution (via PIN mislocalization). This in turn could lead to the vascular patterning defects.

In a second model, it is possible that the defective ethylene responses of *hyd*/*fk* mutants are due to aberrant auxin responses. Auxin itself reinforces PIN localization [58] and can promote ethylene biosynthesis [59], possibly promoting complex feedback effects in the absence of correct sterol profiles. Evidence arguing against this is that the *hyd* mutants do not obviously over-produce ethylene, although the root phenotype of *hyd1* at least can be partially rescued by treatment with the ethylene synthesis inhibitor aminoethoxyvinylglycine [26]. It is therefore possible that the failure of the auxin distribution system in sterol mutants contribute to the observed aberrant ethylene responses, and suppression of the latter by *ein2* in turn ameiorates the auxin transport defects.

Finally, it remains possible that certain sterols are also required as ligands for START domain-containing transcription factors such as PHABULOSA, REVOLUTA, PHAVOLUTA and, indeed, ATHB8 [28], [53], [60], and so have a post-translational role in the function of these proteins in leaf development and vascular differentiation.

It is clear that the phenotypic effects of defective sterol profiles affect multiple signalling pathways that impinge on each other. It would therefore not be appropriate to consider the link between sterol biosynthesis and vascular differentiation as a linear pathway, but rather as a network of interdependent components, the relationships between which we are beginning to unravel. Given the prevalence of feedback loops between auxin, PINs and ethylene [34]–[36], and the implication of sterols as a component in this network, each of the above-mentioned interactions may contribute to the observed complex mutant phenotypes. To date there is no evidence that individual sterols, as distinct from brassinosteroids, act as novel hormone-like molecules. Instead, their main role in development may be via the regulation of cross-talk between established growth regulators such as the auxins and ethylene, through which they modulate the temporal and spatial expression of key regulatory genes.

## Materials and Methods

### Plant material

The *hyd1* and *fk^hyd2^* mutants were identified in a screen of transgenic lines as described previously [21], [26]. *hyd1 ein2-1* and *fk^hyd2^ ein2-1* crosses generated previously as described [33]. For *in vitro* growth studies, *A. thaliana* seeds were stratified and surface sterilized and plated on growth medium (half-strength Murashige and Skoog medium (1/2 MS10; Sigma), 1% sucrose, 3.25 g/l Phytagel agar; Sigma) as described [21].

### Histology

Epidermal cell morphology was revealed by agarose impressions and scanning electron microscopy. To produce agarose impressions, tissue samples were floated on molten 6% (w/v) agarose on the surface of a microscope slide, which was allowed to set before the plant material was removed. The agarose was examined under a light microscope using DIC optics. Leaf material was prepared for visualization of xylem vessels and cleared epidermal cells using standard light microscopy after [61]. To visualize xylem strands, tissues were stained for 5 min with safranin-O (1% w/v in 95% ethanol) and dipped momentarily into 95% ethanol to wash out excess stain, before mounting in 25% (w/v) chloral hydrate. To visualize callose in sieve tube elements in seedlings, aniline blue was used according to [61]. Samples were fixed for 1 hour in 3∶1 ethanol∶acetic acid, cleared overnight in 25% chloral hydrate, and dehydrated through an ethanol series (30%, 50%, 70% v/v for 1 hour each before 96% overnight). After dehydration, leaf tissues were mounted on microscope slides in 50% v/v glycerol prior to microscopy. For visualization of procambial tissue in whole-mount embryos, developing siliques were harvested from plants heterozygous for *hyd/fk* mutations, and carrying the *proAtHB8::GUS* transgene, the testa punctured with a fine tungsten histology needle, and the embryos vacuum-infiltrated with X-Gluc in buffer. GUS-positive embryos were dissected from their seed coat, and mounted in a clearing mixture of 8∶2∶1 (w∶v∶v) chloral hydrate∶glycerol∶water prior to microscopy. The *CYC1At::CDB::GUS* line was fixed in 90% acetone for 15 min on ice prior to incubation, as described [62], to halt cells in the process of dividing. Tissue localization of GUS enzyme activity was performed as described [21].

### Microscopy

For light microscopy, seedlings and embryos were viewed under a Zeiss Axioskop (Carl Zeiss Ltd, Herts, UK). Images were captured as digital images on a Photometrics COOLSNAP™*cf* colour digital camera (Roper Scientific Inc, Trenton, New Jersey, USA) using OpenLab3.1.1 software (Improvision, Coventry, UK). Images were processed in Photoshop 5.0 (Adobe Systems Inc., Mountain View, CA). For GFP analysis, fresh seedlings were mounted in dH_2_O under a large (32×24 mm) zero-thickness coverslip, and examined using either Zeiss LSM510 or Leica SP5 microscopes, argon laser excitation at 488nm and emission filter at 505–530 nm. Images were captured digitally using the integral LSM software. Scanning electron microscopy was carried out as described previously [21].

### Polar auxin transport assays

The polar transport of [^3^H]-IAA (GEH, Amersham, UK) was measured in hypocotyl segments essentially according to [52] and as modified by [63].

## Supporting Information

Figure S1 Correlation between xylem and phloem traces. Merged bright-field and aniline-blue stained UV fluorescence images showing the correspondence between xylem and phloem-associated callose (blue fluorescence) in cotyledons and true leaves. A: Wild-type cotyledon; bar  = 100 µm. B–D: Vascular traces from hyd1 cotyledon (B) and true leaf tissues (C, D). Substantial ectopic callose deposition is found, variably associated with xylem traces; bars  = 100 µm.
(3.70 MB TIF)
