# Supplementary figures and images for: Analysis of Vascular Development in the hydra Sterol Biosynthetic Mutants of Arabidopsis
Source: PLoS One. 2010 Aug 17;5(8):e12227. doi: 10.1371/journal.pone.0012227 (PMC2923191; doi:10.1371/journal.pone.0012227)

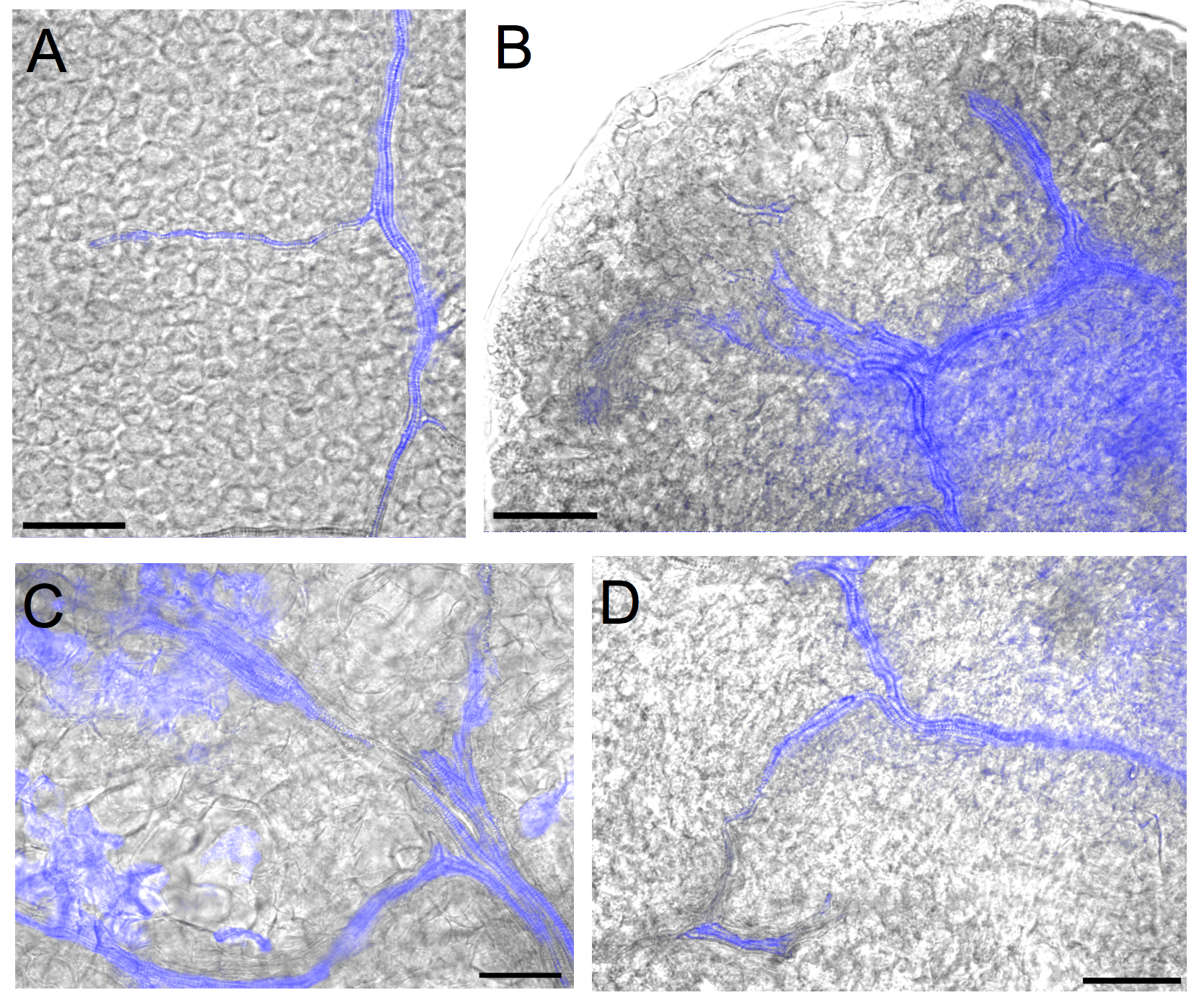

Supplement: Figure S1 — Correlation between xylem and phloem traces. Merged bright-field and aniline-blue stained UV fluorescence images showing the correspondence between xylem and phloem-associated callose (blue fluorescence) in cotyledons and true leaves. A: Wild-type cotyledon; bar = 100 µm. B–D: Vascular traces from hyd1 cotyledon (B) and true leaf tissues (C, D). Substantial ectopic callose deposition is found, variably associated with xylem traces; bars = 100 µm. (3.70 MB TIF) [file pone.0012227.s001.tif]
